# Jordanian Nurses' Perceived Disaster Preparedness: Factors Influencing Successful Planning

**DOI:** 10.1155/2023/5473777

**Published:** 2023-05-16

**Authors:** Mohammad O. Abu Hasheesh

**Affiliations:** Faculty of Nursing, Isra University, Amman, Jordan

## Abstract

**Background:**

Current disaster knowledge, skills, and preparedness levels need to be evaluated to guide plans to strengthen disaster readiness.

**Objective:**

This study aimed to explore the Jordanian staff nurses' perception regarding their familiarity, attitudes, and practices for disaster preparedness (DP) to reduce the negative impacts of disasters.

**Methods:**

This is a cross-sectional, quantitative, descriptive study. The study was conducted on nurses working at governmental and private hospitals in Jordan. A convenience sample of 240 currently working nurses was recruited to participate in the study.

**Results:**

The nurses were somewhat familiar with their role in DP (2.9 ± .84). The nurses' overall attitude towards DP was 2.2 ± 0.38, indicating that respondents had medium attitude levels. A low practice level for DP (1.59 ± 0.45) was also observed. Among the studied demographic variables, there was a significant relationship between experience and prior training with improved familiarity and practices. This indicates a need for strengthening nurses' practical skills as well as their theoretical knowledge. However, there is only a significant difference between attitude scale scores and disaster preparedness training (*f* = 10.120; *p*=0.002).

**Conclusions:**

The study findings support the need for more training (academic and/or institutional) to increase and improve nursing disaster preparedness locally and globally.

## 1. Introduction

A disaster is “a sudden calamitous event that seriously disrupts the functioning of a community or society and causes human, material, and economic or environmental losses that exceed the community's or society's ability to cope using its own resources” [1]. There are two basic sorts of disasters: natural and man-made disasters. Natural disasters include earthquakes, floods, tornadoes, hurricanes, ice storms, volcanic eruptions, tsunamis, and other climatic events, whereas man-made disasters are the result of human activity. Examples of man-made catastrophes include chemical spills, fires, radiological (nuclear) occurrences, biological and chemical terrorism, war crimes, transportation accidents, and explosions [2]. However, according to the sixth anniversary of the adoption of the Sendai Framework, there is no such thing as a natural disaster. It has been emphasized that the global use of fossil fuels, lack of international cooperation to support developing countries and their health systems, environmental destruction, unplanned urbanization, and uncontrolled poverty all increase the frequency and severity of disasters [3]. Hence, there is an urgent need for global collaboration to redefine priorities and act decisively to reduce these existential threats.

Globally, all parts of the world face the possibility of natural and man-made disasters [4]. The range of disasters facing countries around the world is enormous and very diverse, including outbreaks of infectious diseases, unsafe food and water, war and conflicts, extreme weather events, and others [5]. Not surprisingly, these disasters create significant challenges for national governments, because catastrophes cause unimaginable destruction and have an impact on a region's social, political, and cultural conditions [6]. Countries are urged to improve their capacity for health emergency and disaster risk management, integrating measures for prevention, mitigation, readiness, response, and recovery, in order to better handle these calamities and their consequences [7].

Jordan, like other countries in the world, is vulnerable to natural and man-made disasters. Jordan has now been hit by a slew of emergencies [8]. The findings demonstrated that Jordan was under threat from a number of catastrophes and economic insecurity in the Middle East [7]. Terrorism, the Amman bombings in 2005, fire, and floods were also identified as among the emergencies confronting Jordan, having significant consequences for the country's population, infrastructure, and economy [9]. Jordan also faces many climate-related risks and was inundated by heavy rain in October 2018 with two devastating flash floods, killing at least 27 people and evacuating 400 visitors. The first flood washed out a group of teachers and students who were visiting famed hot springs near the Dead Sea, while the second rushed into Jordan's renowned city of Petra [10]. These occurrences have sparked discussions about preparedness, management, and accountability [8–10]. Moreover, Jordan relies heavily on fuel imports, with limited natural resources [11]. Jordan has begun its recovery from the COVID-19 shock [11]. Even before the COVID-19 crisis, Jordan's economy had been struggling with persistently sluggish growth dynamics and structural challenges [12]. Jordan serves as a host country for many United Nations programs in the region. Jordan has experienced part of this in the past decade, including regional conflicts and the influx of nearly 1.3 million Syrian refugees [13].

DP refers to actions made to prepare for and mitigate the effects of disasters [14], that is, to anticipate and, when possible, prevent them to lessen their impact on vulnerable populations, as well as to respond to and cope effectively with their repercussions [15]. DP is a continuous and comprehensive process that involves several actions and resources. It necessitates contributions from a wide range of sectors, beginning with training and logistics and progressing to health care and institutional development [16]. Emergency readiness and a current emergency plan, together with public awareness, will assist society in providing the required materials, as well as adequate training to prevent or reduce hazards [17].

Given the world's rising number of both natural and man-made disasters, educating nurses as the primary group reacting to these incidents is vital [15]. Nurses must be equipped and prepared to react to in catastrophic events. It is known that well preparedness, and the actions one takes during disasters, can have a significant impact on the events' outcomes [18]. Nonetheless, to a great extent, we do not know the Jordanian nurses' attitudes on being prepared to react to these crisis occurrences. This study aims to explore the Jordanian nurses' perception with special consideration to familiarity, attitudes, and practices towards DP and response to mitigate and to reduce their negative impacts.

## 2. Significance of the Study

Nurses make up most of the healthcare workforce, and they have the potential to improve disaster preparation, national capacity, and community resilience to catastrophes. The findings of this study will assist in preparing nursing staff for disaster events. This study will provide insights into the levels of familiarity, attitudes, and practices of Jordanian nurses toward DP. Thus, by being equipped with this knowledge, the processes of DP and risk reduction can proceed more efficiently, resulting in prevention and enhancing response capabilities.

## 3. Purpose of the Study

The purpose of this study was to explore Jordanian nurses' perception with specific regards to familiarity, attitudes, and practices for disaster preparedness.

## 4. Research Questions

This study aimed to answer the following research questions:

1. What is the current level of familiarity of Jordanian nurses for disaster preparedness?
2. What is the current level of attitudes of Jordanian nurses disaster preparedness?
3. What is the current level of practices of Jordanian nurses for disaster preparedness?
4. How do familiarity, attitudes, and practices of Jordanian nurses relate to some demographic variables?

## 5. Methods

### 5.1. Design

This research used a cross-sectional study design, wherein it attempts to assess the Jordanian nurses' perception with special consideration to familiarity, attitudes, and practices towards DP and response to mitigate and reduce their negative impacts.

### 5.2. Setting

The study was conducted in two major government hospitals (Prince Hamzah, with a 436-bed capacity hospital, and Al-Bashir Hospital, providing high-quality care to an estimated 50,000 patients per month in Jordan's busiest public hospital) and three private hospitals (Jordan, Al-Israa, and Specialty) in the central regions of Jordan.

### 5.3. Population and Sampling

The study population participated in this study was staff nurses from various hospitals affiliated to the Ministry of Health and private hospitals in Amman, Jordan, working in different areas of critical and general wards. The convenience sampling approach was used to choose participants who matched the inclusion criteria. Participants were eligible for the study if they met all of the following: participants in the study were those who worked in medical, surgical, critical care, and emergency departments, who were willing to take part, from a variety of age groups, registered nurses, and of both genders. The minimal sample size for the investigation was calculated using the Daniel formula, where *p* is the 50% expectation of the proportion, *z* is the 95% confidence interval, *d* is the error of deviation of 5%, and finally *e* is the determined minimum sample size which was of 220.

### 5.4. The Research Instrument

The instrument used for the data collection contained four parts: demographic variables, nurses' familiarity questionnaire on DP, questionnaire on nurses' attitudes for DP, and questionnaire on nurses' practice for DP. It was developed as a result of an extensive literature review and was consolidated by a pilot study. The first part assessed the respondents in terms of age, gender, years of experience, educational level, and disaster training. The second section included forty statements that assessed nurses' familiarity for DP in nine domains: “detection and respond to event, the incident command system, ethical issues in triage, epidemiology and surveillance, decontamination, communication, psychological issues, special populations, and accessing critical resources.” As a response, a five-point Likert scale was utilized, with one indicating very poor/unfamiliar, two indicating poor/slightly familiar, three indicating fair/familiar, four indicating good/most familiar, and five indicating very good/very familiar. The third section featured 11 questions designed to examine the participants' attitudes toward DP. As a response, a Likert scale was employed, with one indicating disagreement, two suggesting neutrality, and three indicating agreement. The final section of the research instrument was meant to measure participants' practice for DP. As a response, a Likert scale was utilized, with one representing “no,” two suggesting “do not know,” and three indicating “yes.” To assess the questionnaire's validity, professionals in disaster management were asked to rate its relevance. To guarantee consistency, a pilot research was carried out with the goal of attempting to construct the instrument and establishing its reliability using a sample of *n* = 20. Cronbach's alpha test indicated that items were internally consistent at a value of *α* = 0.81.

## 6. Data Collection Procedure

**Figure 1:**
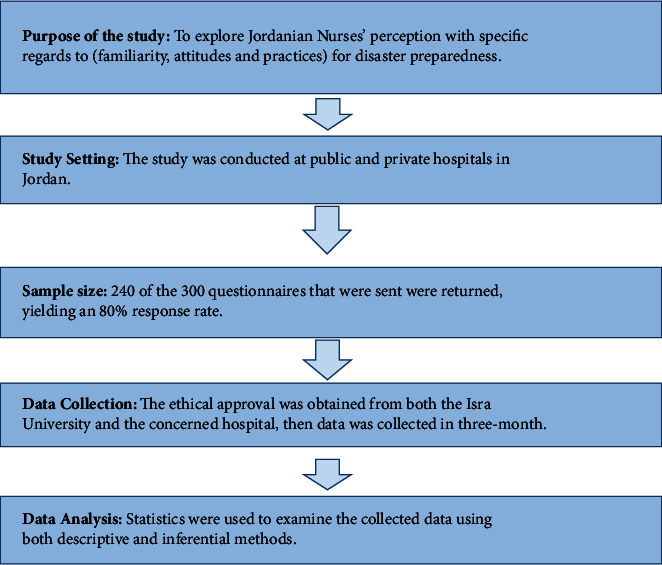
Schematic representation of the research plan (source: Sultan, Mary, and Al Grad, 2017).

The Institutional Review Board (IRB) of Isra University examined and approved this study. The officials of the hospitals were informed of the significance of the study, and permission was obtained to carry it out. The research's goal was described to the nurses, the title and a summary of the study were provided, and verbal consent was obtained from them. They received guarantees that the data gathered from them would be kept confidential and will be solely utilized for the purpose of the study (Figure 1).

## 7. Statistical Methods

Statistics were used to examine the collected data using both descriptive and inferential methods. The demographic characteristics of the study sample were described using percentage distribution and frequency. One-way analysis of variance (ANOVA) was used in this study to examine the individual effects of the independent variables (socio-demographic variables) on the dependent variables (Jordanian nurses' perceptions with special attention to familiarity, attitudes, and practices toward DP). The outcomes of the analysis are highlighted in the following section, along with how they relate to the study's objectives.

## 8. Results

This study aimed to evaluate Jordanian RNs' disaster management readiness. 240 of the 300 questionnaires that were sent were returned, yielding an 80% response rate. With a range of 21–56 years, the mean age was 29.5 ± 6.9 years. Among the participants, there were 118 males (49.2%) and 122 females (50.8%). 170 participants had a bachelor's degree in nursing, while the remaining participants had a nursing diploma. With a range of 1 to 30 years, the experience was 7.4 ± 6.01 years on average. A disaster preparation training activity was attended by around 56.7% of the participants in total, of which 31.9% took part in a training course and 24.8% in a workshop. The demographic findings for the participants are displayed in Table 1.

Table 2 reveals that the research participants' average familiarity score was 2.9 ± 0.84, which is in the “somewhat familiar” range according to the familiarity scale. “Ethical issues in triage” and “the incident command system” had the two highest mean SD familiarity scores 3.13 ± 1.01 and 3.10 ± 1.04, respectively. However, “accessing vital resources” had the lowest mean SD familiarity score of all the dimensions (2.6 ± 1.11). The overall nurses' attitude toward DP, as shown in the table, was 2.2 ± 0.38, suggesting that respondents had moderate attitude levels. Additionally, an inadequate practice level for DP was noted (1.59 ± 0.45).

The result revealed that there is no statistically significant difference between the demographical variables of age and academic qualification with respect to Jordanian nurses' familiarity for DP (*f* = 0.303; *p*=0.739) and (*f* = 1.938; *p*=0.165), respectively. There was also no statistically significant difference between Jordanian nurses' familiarity for DP with gender (*f* = 0.143; *p*=0.706). Meanwhile, regarding training on disaster, F-test yielded statistically significant difference between Jordanian nurses' familiarity for DP and training (*p*=0.000). This implies that participants who attended the training sessions (*m* = 3.21) had better knowledge of DP compared with their colleagues who did not attend such training (*m* = 2.63). Other significant differences were also found between total scores of familiarity scale and experience (*f* = 5.77; *p*=0.004) study variable. Finally, Tukey's posttest revealed significant differences in familiarity score between participants with 5 to 10 years of experience compared to their counterparts with less than five years of experience and over 10 years of experience, indicating that they are more familiar with DP. Perhaps, this is due to the fact that this category is characterized by a desire to learn. It may have participated in courses and workshops on preparedness for disasters, whether through the hospitals in which they worked or through the courses they studied during the bachelor's stage (Table 3).

Table 4 shows the relationship between Jordanian nurses' attitudes for DP and their gender, age, years of experience, qualification, and training on disaster. Regarding (gender, age, experience, and academic qualification), there was no statistically significant relationship between Jordanian nurses' attitudes for DP and their gender, age, years of experience, and academic qualification (*f* = 3.606: *p* = 0.059; *f* = 1.429: *p* = 0.242; *f* = 1.342: *p* = 0.263; *f* = 1.106: *p* = 0.294). Meanwhile, on the relationship between Jordanian nurses' attitudes for DP and their training activity, it showed that there is a significant relationship with a score of *f* = 10.120, *p* = 0.002. That is, participants who attended disaster preparedness training activities (m = 2.36) had better attitudes towards disaster preparedness than their colleagues who did not attend such activities (m = 2.20). This emphasizes the need for training programs on disaster preparedness and management to increase nurses' attitudes towards DP.

**Figure 2:**
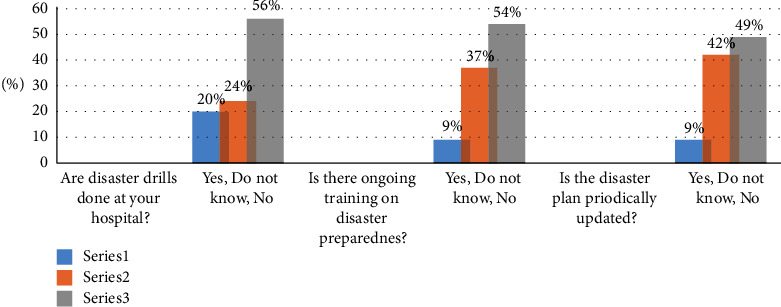
Participants' practice for disaster preparedness.

Table 5 demonstrates results of analysis of variance (*F*) test for significance of differences in practice scale with nurses' profile (gender, age, years of experience, qualification, and training on disaster). The analysis of results shows that there are no significant differences between the variables of gender and academic qualification in relation to total scores of practice scale. However, significant differences exist between scores of practice scale and age, experience, and training on disaster preparedness (*f* = 8.540: *p*=0.000; *f* = 9.024: *p*=0.000; *f* = 42.73: *p*=0.000). Post hoc Tukey's test revealed a significant difference in the practice scores among participants who are more than 35 years old compared with their counterparts less than twenty-five and between 25 and 35 years old; this indicates that they are more likely than their colleagues to be equipped with the practice and knowledge necessary to contribute critical assets to disaster response. Furthermore, the data in Figure 2 show nurses' practice for DP. In particular, for the item that says “are disaster drills done in your hospital?,” the answers were as follows: (20%) yes, (24%) do not know, and (56%) no. As for the other item, “is there ongoing training on DP?,” the answers were (9%) yes, (37%) do not know, and (54%) no. However, regarding the third item in the practice scale which states “are the disaster plans occurring periodically updated?,” the answers were (9%) yes, (42%) do not know, and (49%) no.

## 9. Discussion

The current study revealed the overall familiarity of Jordanian nurses, with the majority of participants rated as somewhat familiar according to the familiarity scale. The lack of disaster preparedness knowledge of Jordanian nurses might be attributed to a lack of efficient training programs during their curricula and working years in hospitals. As a result, nurses are malnourished in terms of crisis management knowledge and abilities. Several studies have revealed that most nurses lack the information necessary for DP, and only a handful can appropriately respond to severe crises [18, 19]. Rabaya et al. discovered that nurses were inadequately knowledgeable with disaster preparation and had to learn more. Their study recommended the need for continuing education [20]. Similarly, research was conducted to analyze the level of readiness of Medina nurses in terms of disaster management, particularly their knowledge. The study found an average degree of readiness for disaster management among Saudi nurses and advised that they should receive sufficient education about Emergency Preparedness Information Questionnaire (EPIQ) dimensions [21]. According to research, medical records employees in hospitals and other healthcare professionals generally have inadequate levels of expertise [22, 23]. However, in a study conducted in the Najran region of Saudi Arabia, the participating respondents indicated good knowledge (familiar to very familiar) in almost all investigated areas of the conceptual underpinnings of emergencies, including emergency preparedness activities, incident command systems and their function, ethical considerations in triage, epidemiology and surveillance, isolation and quarantine, decontamination, communication considerations, psychological considerations, management of special/vulnerable populations, and assessment of critical resources [24]. Many knowledge, attitude, and practice (KAP) studies on the role of nurses in disaster and emergency preparedness have found that while nurses are aware of the necessity for such preparation, they are not sufficiently prepared for catastrophes [21–23]. Consequently, nurses' fundamental education should incorporate disaster management training.

As for Jordanian nurses' attitudes toward DP, according to the results of the current study, the general nurses' attitude toward DP was 2.2 ± 0.38, which is considered to be a medium attitude level. Among the research studies conducted in this area, Abdelghany Ibrahim et al. found a lack of behaviors and knowledge along with an acceptable degree of attitude toward disaster preparedness and an awareness with emergency preparedness that was neutral [25]. However, Yousefi et al. found that Iranian nurses had positive attitudes, moderate levels of knowledge and competence, and readiness for catastrophes [26]. Several studies have found a positive association between knowledge and attitude, and knowledge is seen to be a favorable predictor of desired attitude [23, 25, 27]. This means that increased knowledge affects the nurses' attitudes regarding disasters, and as nurses' knowledge increases, they will understand disasters better and try to remove existing obstacles. Furthermore, being the biggest subset of healthcare workers, nurses must have enough information and knowledge and be prepared to demonstrate the disaster response. In fact, nurses require sufficient knowledge and skills through operational maneuvers and continuing education to achieve the appropriate attitude regarding DP.

With respect to nurses' practice toward DP, the overall practice of the study participants was insufficient. This may be due to that educational programs in Jordan and hospitals do not adequately address the needs of nursing students and nurses for DP. A similar finding was concluded by Singhal et al. on the basis of their quantitative study that participants' level of practice was negative in relation to disaster preparedness [28]. The findings are also in agreement with Abdelghany Ibrahim et al. who discovered that nurse practice-related disaster preparedness was below average [25]. However, a study conducted by Putra et al. reported that public health nurses' ability in practical dimensions was at a moderate to high level, and they were willing to participate in disaster preparation programs [29]. As a result, offering structured and frequent training to nurses is critical for expanding their knowledge and abilities during both their educational and working years [30–32].

## 10. Conclusion

The results indicate that the Jordanian nurses were somewhat familiar with their role in DP and had a medium attitude level for DP; but, an insufficient practice level for DP was also observed, because catastrophe familiarity and information must be combined with practical skills in order to obtain good outcomes in disaster occurrences. As a result, disaster planning, increased training, and enhancing practical emergency preparation exercises, such as scenario-based simulation exercises, may improve nurses' readiness for disaster response.

## 11. Implications

The current study has several implications for nurses during a disaster. One major purpose of the nurses' work during a crisis is to help as many people as possible with their health. To do so, the nurse must be ready to help at all stages of a crisis. During a crisis, nurses must undertake a variety of jobs and difficulties, such as triaging patients, allocating nursing personnel effectively to provide effective treatment, ensuring equipment and supplies are distributed correctly, and supervising patient transfers to hospitals. The line of command throughout a crisis is an important feature that nurses must understand. Furthermore, a written plan has to be in place before the disaster, with clear roles that are understood by all nursing staff, and the individual selected to be the nurse leader must be identified and his/her roles clearly defined. Hence, we may be able to return and work quickly and start serving the people affected by the disaster.

## 12. Recommendation

Findings of the current study indicated that nurses' are somewhat familiar with their role in DP, their attitudes are moderate, and their practical skills are insufficient. Hospital nurses still have a lot to learn about disaster preparation. It is suggested that ongoing education be pursued. Future studies might look at the link between knowledge and attitude or knowledge and practice.

## 13. Limitation

One of the limitations of the study is convenient sampling, whereas the probability sampling approach can improve the induction of diverse strata of participants. Another weakness of this study is its cross-sectional design.

## Figures and Tables

**Table 1:** Demographic profiles of the study sample.

Variable	Number (%)/*M* ± SD
Age	
Less than 25	68 (28.3)
25–35	132 (55)
More than 35	40 (16.7)

Gender	
Male	118 (49.2)
Female	122 (50.8)

Nursing experience	
1–5 year	38 (15.8)
6–10 year	150 (62.5)
More than 10 year	52 (21.7)

Education	
Bachelor	170 (70.8)
Diploma	70 (29.2)

Training on disaster	
Yes	136 (56.7)
No	104 (43.3)

Type of training	
Course	76 (31.9)
Workshop	60 (24.8)
Total	136 (56.7)

*N* = 240.

**Table 2:** Familiarity, attitudes, and practices of Jordanian nurses for disaster preparedness.

Questionnaire	Domain	Frequency	Mean	±SD
Familiarity	Detection and response to event	240	2.80	0.88
	The incident command system	240	3.10	1.04
	Ethical issues in triage	240	3.13	1.01
	Epidemiology and surveillance	240	2.7	1.08
	Decontamination	240	3.01	1.13
	Communication	240	2.99	0.95
	Psychological issues	240	2.9	1.02
	Special populations	240	2.9	1.06
	Accessing critical resources	240	2.6	1.11
	Total	240	2.9	0.84

Attitudes	Total	240	2.2	0.38

Practices	Total	240	1.59	0.45

*N* = 240.

**Table 3:** Jordanian nurses' familiarity for disaster preparedness across their profiles.

Variable	Category	Frequency	Mean	±SD	*F*	*p* value
Gender	Male	118	2.88	0.86	0.143	0.706
	Female	122	3.04	0.83		

Age	>25	68	2.90	0.81	0.303	0.739
	25–35	132	3.00	0.79		
	<35	40	2.92	1.08		

Experience	>5	38	2.69	0.61	5.77	0.004
	5–10	150	3.08	0.85		
	<10	52	2.81	0.94		

Qualification	Diploma	70	3.15	0.66	1.938	0.165
	Baccalaureate	170	2.88	0.90		

Training activity	Yes	136	3.21	0.73	30.99	0.000
	No	104	2.63	0.88		

Total		240	2.96	0.85		

Note. ^*∗*^The mean difference is significant at the 0.05 level.

**Table 4:** Jordanian nurses' attitudes for disaster preparedness across their profiles.

Variable	Category	Frequency	Mean	±SD	*F*	*p* value
Gender	Male	118	2.22	0.39	3.606	0.059
	Female	122	2.35	0.37		

Age	>25	68	2.24	0.39	1.429	0.242
	25–35	132	2.34	0.32		
	<35	40	2.20	0.53		

Experience	>5	38	2.30	0.18	1.342	0.263
	5–10	150	2.32	0.39		
	<10	52	2.19	0.48		

Qualification	Diploma	70	2.36	0.18	1.106	0.294
	Baccalaureate	170	2.26	0.44		

Training activity	Yes	136	2.36	0.34	10.120	0.002
	No	104	2.20	0.42		

Total		240	2.29	0.39		

Note. ^*∗*^ The mean difference is significant at the 0.05 level.

**Table 5:** Jordanian nurses' practices for disaster preparedness across their profiles.

Variable	Category	Frequency	Mean	±SD	*F*	*p* value
Gender	Male	118	1.60	0.46	2.66	0.104
	Female	122	1.60	0.44		

Age	>25	68	1.49	0.41	8.540	0.000
	25–35	132	1.58	0.45		
	<35	40	1.81	0.45		

Experience	>5	38	1.56	0.56	9.024	0.000
	5–10	150	1.55	0.47		
	<10	52	1.74	0.38		

Qualification	Diploma	70	1.57	0.41	0.577	0.448
	Baccalaureate	170	1.61	0.47		

Training activity	Yes	136	1.67	0.48	42.73	0.000
	No	104	1.51	0.40		

Total		240	1.59	0.45		

Note. ^*∗*^The mean difference is significant at the 0.05 level.

## Data Availability

All data generated or analyzed during this study are included in this article. The data sets used and/or analyzed during the current study are available from the corresponding author on reasonable request.
